# 441. The Effects of Race and Comorbidity Burden on Inflammatory Biomarkers Among Persons Hospitalized with COVID-19

**DOI:** 10.1093/ofid/ofab466.640

**Published:** 2021-12-04

**Authors:** Yetunde A Fatade, Lauren F Collins, Lauren F Collins, Zakaria Almuwaqqat, ZhenChao Chen, Mahadev Prasad, Arshed Quyyumi, Igho Ofotokun

**Affiliations:** 1 Emory University, Snellville, GA; 2 Division of Infectious Diseases, Emory University School of Medicine, Atlanta, GA

## Abstract

**Background:**

African Americans (AA) and Latinos, compared with Whites, experience disproportionately higher rates of morbidity and mortality in COVID-19. Exuberant inflammatory responses may explain, in part, the differences in disease severity in COVID-19 observed among different demographic groups.

**Methods:**

In a retrospective cohort study, we analyzed data from patients aged ≥18 years hospitalized for COVID-19 (confirmed by positive SARS-CoV-2 PCR) from 3/1/2020 – 12/31/2020 at Emory Healthcare hospitals. Patient demographics, clinical characteristics, and peak levels of high-sensitivity C-reactive protein (hs-CRP) during hospitalization were abstracted from electronic medical record. Comorbidity burden was defined as the number of six total comorbidities assessed per patient. Multivariable logistic regression (adjusted for age, sex, body mass index [BMI], smoking status) assessed the effects of race and comorbidity burden on peak hs-CRP level.

**Results:**

3,860 patients, median age 60 [18-108] years, 51% female, 57% AA, 28% White, 6% Latino and 9% other races were enrolled. Median comorbidity burden per patient was 2 (Q1-Q3, 1-3), with prevalent comorbidities distributed as follows: 68% had hypertension, 43% renal disease, 42% diabetes, 16% cardiovascular disease, 12% lung disease, and 5% cancer. Unadjusted peak hs-CRP (mg/L) levels were highest among Latino patients (144.9) followed by other races (137), AA (130.3), and Whites (122.2). In adjusted models (including race), the mean difference in peak hs-CRP (mg/L) compared with patients who had no comorbidities was 18.7 (p=0.108), 56.7 (p< 0.001), and 78.2 mg/L (p< 0.001) for 1, 2, and ≥3 comorbidities, respectively. In adjusted models (including comorbidity burden), the mean level of peak hs-CRP, compared with Whites, was 34.2 (p< 0.001), 38.4 (p=0.003), and 36.0 mg/L (p=0.06) higher in AA, Latinos, and other races, respectively.

**Conclusion:**

Among patients hospitalized with COVID-19, non-White race and comorbidity burden were associated with significantly higher levels of inflammation. These findings suggest that exuberant inflammatory responses may be driving, in part, the differences in COVID-19 disease severity observed across different demographic groups.

**Disclosures:**

**Lauren F. Collins, MD, MSc**, Nothing to disclose

